# Biotransformation studies of textile dye Remazol Orange 3R

**DOI:** 10.1007/s13205-012-0093-1

**Published:** 2012-10-06

**Authors:** Swati V. Surwase, Krutika K. Deshpande, Swapnil S. Phugare, Jyoti P. Jadhav

**Affiliations:** 1 Department of Environmental Biotechnology, Shivaji University, Kolhapur, 413007 India; 2 Department of Biochemistry, Shivaji University, Kolhapur, 413007 India; 3 Department of Biotechnology, Yashwantrao Chavan College of Science, Vidyanagar, Karad, 415124 India

**Keywords:** Biodegradation, Remazol Orange 3R, Tyrosinase, HPLC, GC–MS

## Abstract

In the present study, biotransformation of Remazol Orange 3R (RO3R) was studied using well-known bacterial isolate *Pseudomonas aeruginosa* strain BCH. The dye was decolorized up to 98 % within 15 min. The induction in the level of various oxidoreductive enzymes viz. laccase, tyrosinase, veratryl alcohol oxidase and DCIP reductase were observed in the cells obtained after decolorization of RO3R, which supports their role in decolorization. The metabolites of RO3R obtained after biodegradation were identified and characterized by various analytical techniques viz, HPLC, FTIR, and GC–MS. The RO3R was transformed to the *N*-(7 amino 8 hydroxy-napthalen-2yl) actamide (m/z, 198), Acetamide (m/z, 59) and Napthalen-1-ol (m/z, 144).

## Introduction

The colors are inseparable part of human life and gives delightful pleasures to the eyesight. On the other hand, colors are responsible for severe environmental pollution in the present date. The textile, pharmaceutical, tattooing, cosmetics and food industries utilizes thousands of synthetic azo dyes (Kolekar and Kodam 2011). The studies showed that, numbers of dyes are released as waste product from such industries. These dyes or their degradation products, when released into water even at very low concentrations can be toxic and sometimes carcinogenic, mutagenic, or teratogenic to various organisms, including humans (Novotny et al. 2006; Hai et al. 2007). Large amounts of the dyestuff, which are usually unbound dye materials, are directly lost to the wastewater, during textile processing (Gomare et al. 2008). In the present scenario, the release of colored wastewater to the ecosystem is major source of eutrophication and perturbations in aquatic life. The discharge of such effluents from textile industries can result into serious environmental damage. The textile dyes especially azo dyes are known to be xenobiotic compounds and often reported to be recalcitrant to the biodegradation processes. The color removal is of prime importance because of toxicity of dyes and dye effluents to aquatic life, mutagenicity of azo dyes, and carcinogenicity of their degradation products (Rajaguru et al. 2000). Considering the overall load of effluents from the textile industries, dye wastewaters are usually treated using physicochemical methods (Brent et al. 2006; Hasnat et al. 2007). The physical and chemical methods are highly expensive, coupled with the formation of large amount of sludge and the emission of toxic substances (Johnson et al. 1978). In addition, the accumulation of concentrated sludge creates a disposal problem (Banat et al. 1996).

Biological treatment of dye wastewater is one of the alternative method and currently gaining much importance as an expanding technology. The cost-effectiveness, lower sludge production, and ecological sociability of biological systems have made them more favorable, compared to physicochemical methods, for the treatment of textile-printing wastewater (Dawkar et al. 2010). Bioremediation process involves the improvement of natural degradation capacity of the microorganism (Kalyani et al. 2008). Several researchers have reported decolorization of various textile dyes using microorganisms including bacteria and fungi. Majority of research articles focuses on decolorization of azo dyes by various microorganims (Manu and Chaudhari 2003; Maximo et al. 2003; Beydilli and Pavlostathis 2005). Various *Pseudomonas* species were reported for decolorization of sulfonated azo dyes by under static conditions (Banat et al. 1996; Puvaneshwari et al. 2002). Remazol Orange 3R is sulfonated azo dye and commonly used in the textile processing. Metabolic fate of RO3R was previously reported using plant species consortium (Kabra et al. 2011). In the present study, focus is given for the biotransformation studies of sulfonated azo dye RO3R using bacterial strain *Pseudomonas aeruginosa* strain BCH.

## Materials and methods

### Microorganism and chemicals

The bacterial strain used in the present study was previously isolated in our laboratory from the dye-contaminated sludge (Jadhav et al. 2010). The strain was identified as *Pseudomonas aeruginosa* strain BCH by 16S rRNA gene sequence analysis and the sequence is deposited in the GenBank database under the accession number FJ 496659.

Nitro-blue tetra zolium (NBT) and riboflavin were obtained from Hi-media, Mumbai, India. Textile dye RO3R (Color index number -17757, purity ≥70 %) was a generous gift from local textile industry, Ichalkaranji, India. All the other chemicals used in the present study were of an analytical grade and highest purity available.

### Culture conditions

The strain was maintained routinely on yeast extract agar medium having composition (g l^−1^); yeast extract (5), NaCl (5), agar (25). Decolorization studies were performed in yeast extract medium having same composition as stated above except agar. All the decolorization experiments were carried out at 30 °C, under static conditions unless otherwise stated.

### Decolorization of studies

The RO3R was added at the concentration of 50 mg l^−1^ to 250 ml Erlenmeyer flask containing 100 ml bacterial growth (24 h grown). Aliquots (3 ml) of the culture media were withdrawn at different time intervals and centrifuged at 7,000*g* for 15 min to separate the bacterial cell mass. Decolorization of the dye RO3R was analyzed using UV–Vis spectrophotometer (Hitachi U 2800, Tokyo, Japan) at 470 nm. All decolorization experiments were performed in triplicate and the decolorization activity was expressed in terms of the percentage decolorization using following formula:

The above-mentioned protocol was followed while studying the effect of static and shaking conditions, physico-chemical parameters, increasing dye concentration, effect of carbon, and nitrogen sources on decolorization.

### Acclimatization

Acclimatization was performed by gradually exposing *Pseudomonas aeruginosa* strain BCH to increasing dye concentrations as reported earlier (Kalme et al. 2006; Kalyani et al. 2008). *Pseudomonas aeruginosa* strain BCH was consecutively transferred into the nutrient agar medium with increasing concentration of RO3R dye up to 1,500 mg l^−1^ at 30 °C and static condition. Acclimatized strain was routinely maintained on dye agar slant and used for further experiments.

### Preparation of cell-free extracts

The bacterial cells were harvested after 24 h by the centrifugation at 7,000*g* for 20 min and considered as a control. The cell pellets were suspended in 50 mM potassium phosphate buffer (pH 7.4) and sonicated (Sonics-vibracell ultrasonic processor) by giving seven strokes, each of 60 amplitude for 30 s with 2-min time interval between two strokes. Sonication was performed at 4 °C. The sonicated cells were centrifuged in cold condition (at 4 °C; 7,000*g* for 20 min) and used as the source of intracellular enzymes. Similar procedure was used to quantify the enzyme activities after dye decolorization.

### Enzymatic assays

Activities of laccase, veratryl alcohol oxidase, tyrosinase and DCIP reductase were assayed spectrophotometrically. The laccase and veratryl alcohol oxidase assays were performed as reported earlier (Jadhav et al. 2011). DCIP reductase was calculated by previously quoted method (Salokhe and Govindwar 1999). The reduction of DCIP was calculated using extinction coefficient of 19 mM cm^−1^. Tyrosinase activity was calculated by using previously reported protocol (Ali et al. 2007).

### Biodegradation analysis

The culture broth after RO3R decolorization was extracted with equal volume of ethyl acetate. The extracts were then dried by evaporation; small portion of remaining residue was redissolved in HPLC grade methanol and used for, HPLC and GC–MS analysis whereas remaining residue was mixed with stereoscopically pure KBr and used for FTIR analysis. FTIR analysis was carried out using Simadzu 8400S spectrophotometer in the mid-infrared region of 400–4,000 cm^−1^ with 16-scan speed. HPLC analysis was performed in an isocratic Waters 2690 system equipped with dual absorbance detector, using C_18_ column (4.6 × 250 mm) and HPLC grade methanol and water (90:10) as mobile phase with flow rate 1 ml min^−1^. The identification of metabolites formed after degradation of RO3R was carried using a QP2010 gas chromatography coupled with mass spectroscopy (Shimadzu, Japan). The ionization voltage was 70 eV. Gas chromatography was conducted in the temperature programming mode with a Restek column (0.25 mm, 60 m; XTI-5). The initial column temperature was 80 °C for 2 min, then increased linearly at 15 °C min^−1^ to 210 °C followed by 10 °C min^−1^ increase up to 240 °C and held for 2 min, finally giving 5 °C min^−1^ rise up to 280 °C and held for 10 min. The temperature of the injection port was 280 °C and the GC/MS interface was maintained at 290 °C. The helium carrier gas flow rate was 1.0 ml min^−1^. Metabolites were identified using NIST library assisted with MS engine.

### Statistical analysis

Data were analyzed by one-way analysis of variance (ANOVA) with the Tukey–Kramer multiple comparisons test.

## Results and discussion

### Decolorization analysis under various physico-chemical conditions

**Fig. 1:**
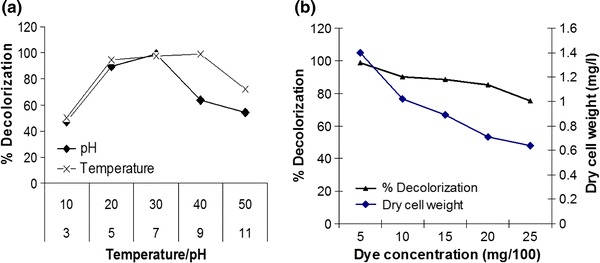
Effect of temperature and pH on decolorization of Remazol Orange 3R (**a**); effect of initial dye concentration on decolorization of Remazol Orange 3R (**b**)

The bacterial strain *Pseudomonas aeruginosa* strain BCH was tested for decolorization of various dyes and among the tested dyes, RO3R was found to be decolorized within 15 min with 98 % decolorization efficiency. The present study reports fastest decolorization and efficient of RO3R. *Pseudomonas aeruginosa* strain BCH is well-known strain for faster decolorization of textile dyes (Jadhav et al. 2010; 2011). The strain *Pseudomonas* sp SUK is one of the efficient bacterial strain reported for faster decolorization of various textile dyes including Reactive blue 59, Reactive Yellow 81, Reactive Red-Brown with in 2, 4 and 4 h, respectively (Kalyani et al. 2009). Decolorization of Methyl Red was reported within 16 min by *Saccharomyces cerevisiae MTCC 463* (Jadhav et al. 2007). Previously phytodecolorization of RO3R was reported with 100 % decolorization with 36 h by a plant consortium (Kabra et al. 2011). It was found that change in pH affects the decolorization rate (Fig. 1a). *Pseudomonas aeruginosa* showed significant growth at pH 5, 7, and 9 but highest decolorization (98.88 %) was observed at pH 7. Decolorization was found to be up to 89 and 93 % at pH 5 and 9, respectively; while pH 3 and 11 were found to be inhibitory for the growth ultimately reducing the decolorization rate. It was noted that change in the temperature significantly affected the decolorization rate (Fig. 1a). The optimum decolorization was recorded at 40 °C (98.89 %), for 10 and 50 °C decolorization was noted to be, 50 and 71 %, respectively. It was noted that far more increase or decrease in temperature affects decolorization rate. Studies showing the effect of various environmental parameters on the decolorization rate was reported previously in case of Direct Orange 39 decolorization (Jadhav et al. 2010). All the decolorization experiments were performed under static conditions as the decolorization was not favored under shaking conditions. Similarly, decolorization was not observed in sterile cell-free medium, indicating none of the abiotic factor was involved in the decolorization process. The decolorization was not observed in case of heat-inactivated cells, indicating that the decolorization was mainly attributed by the metabolic activity of the bacterial cells and not by physical adsorption. The physicochemical parameters may affect the stability of enzyme system involved in dye degradation, resulting in decreased performance in decolorization activity at extreme pH and temperature, which may affect the viability of strain (Jadhav et al. 2011).

### Effect of increasing dye concentration

The Fig. 1b showed the influence of initial dye concentration on decolorization of RO3R. The decolorization efficiency decreased when the concentration increased from 50 to 2,500 mg l^−1^. Increase in dye concentration resulted in a significant change in percentage decolorization as well as the time required for decolorization. Optimum decolorization was observed to be 98 % for 50 mg l^−1^ of dye concentration within 15 min. The time required for decolorization was gradually increased up to 45 min for 2,500 mg l^−1^ with decolorization efficiency reduced to 85 %. The results indicated that increase in dye concentration might be affecting overall growth and enzyme systems involved in decolorization of RO3R, ultimately resulting into the decrease decolorization rate. Similar studies were reported earlier for biodegradation of reactive yellow 84A where *Exiguobacterium* sp. RD3 could decolorize the dye maximally up to 1 g l^−1^ with 21.05 % decolorization efficiency (Dhanve et al. 2008).

### Effect of carbon and nitrogen sources on decolorization

**Fig. 2:**
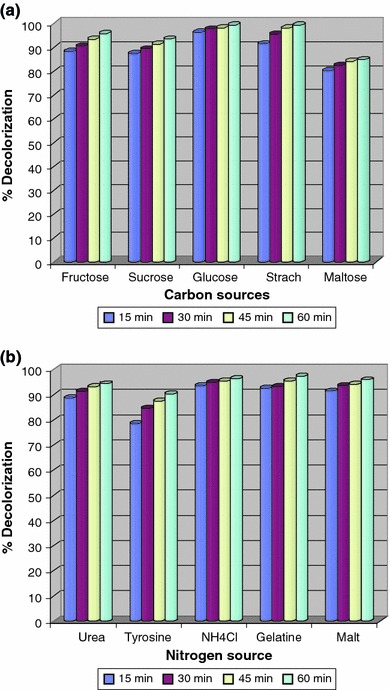
Effect of various carbon (**a**) and nitrogen (**b**) sources on decolorization of Remazol Orange 3R

The growth of the microorganism mainly depends up on the nutrient availability in the growth habitat. All metabolic activities of the microbes are regulated through the nutrient status of the medium. Under starvation microbes switch on the alternative metabolic pathways to utilize the available material as a food source. Sometimes additional carbon and nitrogen sources act as a stimulator for metabolic activity. In the presence of different carbon and nitrogen sources the decolorization ability was reduced. Glucose and starch was found to stimulate decolorization. It was noted to be around 99 % of decolorization was achieved when the medium was supplemented with additional glucose and starch, which might be due induction in the growth of microorganism. Effect of various carbon sources on decolorization performance is as summarized in Fig. 2a. Glucose may enhance decolorization by allowing the faster growth of actively respiring bacteria resulting in rapid depletion of oxygen from the medium and thus creating the conditions favorable for anaerobic reduction of azo dyes (Haug et al. 1991). However, nitrogen sources dose not seemed to contribute much to the decolorization performance of the bacterial strain. Effect of various nitrogen sources on decolorization of RO3R is as summarized in Fig. 2b. It was found that yeast extract itself served efficient nitrogen source for the *Pseudomonas aeruginosa* strain BCH.

### Effect of salinity on decolorization

The *Pseudomonas aeruginosa* strain BCH showed significant growth and decolorization, up to 5 % of salt concentration. As the salt concentration increased above 5 % overall growth rate and decolorization efficiency was also reduced. Salt concentration above 5 % was found to inhibit the growth. It was noted that for 1 % salt concentration the decolorization was near about 87 %, which was less as compared to the decolorization in normal medium. At higher salinity as, 5 % of salt concentration the decolorization rate was dropped down to 79 %. The *Pseudomonas aeruginosa* strain BCH was previously reported for dye decolorization at higher salt concentration (Jadhav et al. 2010). The ability of the strain to tolerate salt concentration up to 5 % makes it significant, as it can decolorize RO3R, at high salinity levels also. Textile waste also contains various salts along with dyes; hence *Pseudomonas aeruginosa* strain BCH could be effective candidate for textile wastewater treatment. High salt concentration may cause inhibition of microorganisms and eventually the loss of activity of cell. Generally, sodium concentration above 3 g l^−1^ can cause moderate inhibition of most bacterial activities (De Baere et al. 1984). Inhibition to microorganisms by high salt concentration may cause plasmolysis or loss of activity of cells (Panswad and Anan 1999).

### Enzymatic analysis

Since the enzyme used in present study is in crude form, it highlights the combined action of studied oxidative and reductive enzymes during biodegradation of RO3R. Enzymatic studies showed increase in the activities of laccase, veratryl oxidase, tyrosinase, and DCIP reductase. Significant increase in the activities of these enzymes, after decolorization, indicates involvement of these enzymes for degradation of RO3R. The combined action of these oxidoreductive enzymes might be responsible for biodegradation and decolorization of RO3R. Table 1 represents the enzyme activities before (control) and after treatment of RO3R with *P. aeruginosa* BCH. The involvement and role of these oxidoreductive enzymes during dye decolorization is well documented (Telke et al. 2009; Jadhav et al. 2010).

**Table 1:** Enzyme activities in cells of *Pseudomonas aeruginosa* BCH before and after Remazol Orange 3R decolorization

Enzyme activity	Before dye decolorization	After dye decolorization
Laccase^a^		
Intracellular	0.296 ± 0.098	0.109 ± .056
Extracellular	0.076 ± 0.008	0.379 ± 0.045**
Veratryl alcohol reductase^a^	6.5 ± 0.56	8.58 ± 0.89*
DCIP reductase^b^	12.34 ± 0.94	18.54 ± 1.34**
Tyrosinase^a^	0.054 ± 0.004	1.88 ± 0.57**

Values are the mean of three experiments, ±SEM. Significantly different from control cells at * P < 0.05; ** P < 0.001 by one-way analysis of variance (ANOVA) with Tukey–Krammer comparison test

^a^Enzyme units mg^−1^ min^−1^

^b^μg of DCIP reduced min^−1^ mg^−1^ protien

### Biodegradation analysis

**Fig. 3:**
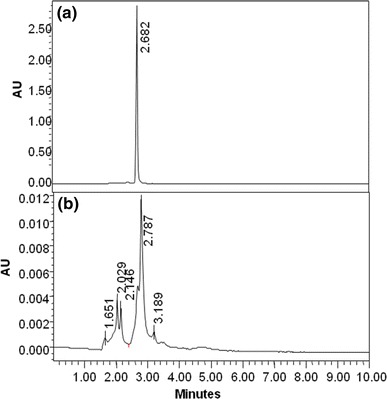
HPLC elution profiles of, dye Remazol Orange 3R (**a**), biodegradation metabolites of Remazol Orange 3R (**b**)

HPLC, FTIR and GC–MS analysis helped us to investigate the RO3R dye degrading mechanism of *Pseudomonas aeruginosa* strain BCH. The HPLC analysis of control dye sample showed a single peak at retention time 2.6 min (Fig. 3a). As, the decolorization progressed the emergence of additional peaks were observed due to degradation of parent dye at retention time 1.6, 2.4, 2.7, and 3.1 min (Fig. 3b). The biodegradation of RO3R is well supported with the help of HPLC analysis, as not only change in peak pattern was observed but also corresponding decrease in absorbance was also noted, which concludes decrease in the concentration of dye sample. The dye decolorization and degradation was supported earlier with help HPLC analysis by various researchers (Kalyani et al. 2008; Telke et al. 2009; Phugare et al. 2011).

**Fig. 4:**
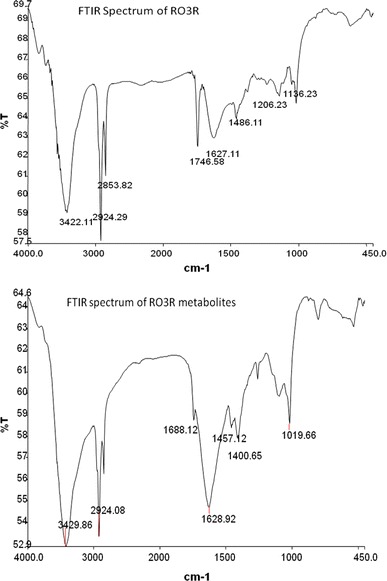
FTIR spectrum of Remazol Orange 3R, biodegradation metabolites of Remazol Orange 3R

The FTIR spectrum of RO3R dye (Fig. 4) showed presence of different peaks at 3,422 cm^−1^ for N–H stretching of secondary amides, 2,924 cm^−1^ for C–H stretching of alkanes, 1,627 cm^−1^ for N=N stretching of as in azo compounds, 1,486 cm^−1^ for N–H trans stretching for secondary amides, 1,206 cm^−1^ for S=O stretching of sulfonic acid compounds, and 1,136 cm^−1^ for S=O asymmetric stretching of sulfones. The FTIR spectrum of RO3R dye metabolites (Fig. 4) showed major peaks at 3,429 cm^−1^ for N–H starching of secondary amides, free NH, 2,924 cm^−1^ for C–H stretching of alkanes, 1,688 cm^−1^ for acetamide, which supports removal of acetamide molecule by microbial action, which can be supported with help of GC–MS analysis. Peaks at frequency 1,400 and 1,457 cm^−1^ represents for N–H of stretching of primary amines and C–H stretching of alkanes. The change in peak pattern as well as disappearance of few peaks in samples after degradation showed that biodegradation of the dye has occurred. Similar observations were previously quoted by Kabra et al. (2011) for decolorization of RO3R using plant species *A. amellus* and *G. pulchella.*

**Fig. 5:**
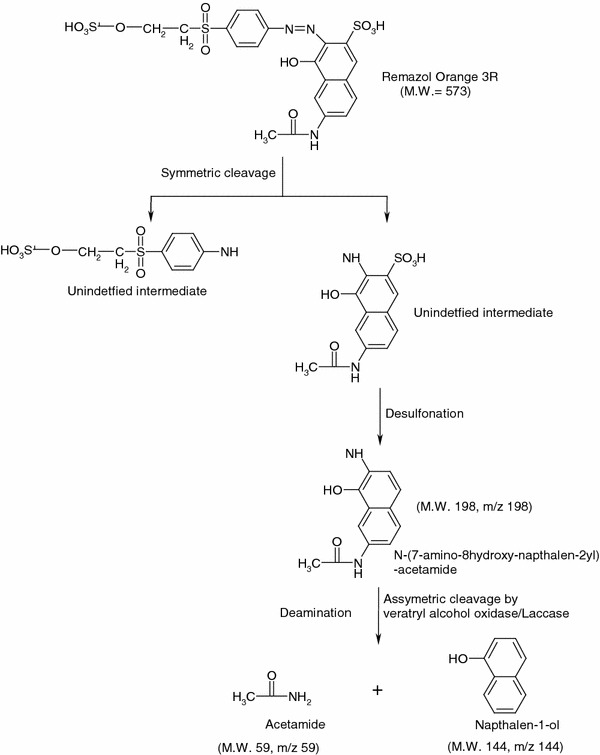
Proposed biodegradation pathway of Remazol Orange 3R

The GC–MS analysis results were used to determine the probable metabolites of RO3R generated after treatment with *Pseudomonas aeruginosa* strain BCH. Azo dyes are cleaved symmetrically or asymmetrically with active site available for an enzyme to excite the molecule (Kabra et al. 2011). Initially, the dye RO3R undergoes symmetric cleavage leading to the formation of two unidentified intermediates, by the action of oxidative enzymes. One of the intermediate further undergoes desulfonation reaction leading to the formation of N (7-amino-8-hydroxy-napthalen-2yl) acetamide. *N*-(7-amino-8-hydroxy-napthalen-2yl) acetamide further cleaved asymmetrically by oxidative enzyme system, followed by deamination reaction which results formation of free acetamide and Napthalen 1-ol. The detailed proposed pathway of RO3R degradation is summarized in Fig. 5. The mass fragmentation pattern of different metabolites is given in the Table 2 to support the biodegradation pathway. Previously, consortium of plants *Aster amellus Linn* and *Glandularia pulchella* (Sweet) *Tronc* was reported for complete decolorization of Remazol Orange 3R in 36 h, while individually *A. amellus* and *G. pulchella* took 72 and 96 h, respectively (Kabra et al. 2011). Remazol Orange 3R was reported to be transformed to different metabolites (acetamide, benzene, naphthalene, 3-diazenylnaphthalene-2-sulfonic acid, 3-diazenyl-4-hydroxynaphthalene-2-sulfonic acid, naphthalen-1-ol) by the plant systems (Kabra et al. 2011). The metabolites produced by plant system and bacterial system are significantly different from each other which concludes different systems follows different degradation mechanism for same dye molecule.

**Table 2:** GC-MS spectral datasheet of metabolites formed after degradation of Remazol Orange 3R

S. no.	Molecular weight of metabolite (m/z)	Retention time (min)	Name of metabolite	Mass peaks
1	144	21.86	Naphthalene-1-ol	
2	198	23.12	*N*-(7-amino-8-hydroxyl-napthalen-2yl)-acetamide	
3	59	12.23	Acetamide	

## Conclusion

The bacterial strain *Pseudomonas aeruginosa* strain BCH bears significant potential to effectively decolorize and transform RO3R with very short period of time to different metabolites. The strain bears faster RO3R decolorizing potential compared previously reported resources. The strain *Pseudomonas aeruginosa* strain BCH could be promising candidate for the RO3R containing textile waste treatment.
